# Editorial: Physiological and Molecular Aspects of Plant Rootstock-Scion Interactions

**DOI:** 10.3389/fpls.2022.852518

**Published:** 2022-02-18

**Authors:** Rosario Paolo Mauro, Francisco Pérez-Alfocea, Sarah Jane Cookson, Nathalie Ollat, Alessandro Vitale

**Affiliations:** ^1^Dipartimento di Agricoltura, Alimentazione e Ambiente (Di3A), University of Catania, Catania, Italy; ^2^Centro de Edafología y Biología Aplicada del Segura (CEBAS), Spanish National Research Council (CSIC), Murcia, Spain; ^3^EGFV, Bordeaux Sciences Agro, INRAE, Univ. Bordeaux, ISVV, Villenave d'Ornon, France

**Keywords:** scion, rootstock, grafting, plant physiology, molecular interactions

## Introduction

Increasing world population, global warming and the depletion of natural resources pose serious threats to food security for future generations, both from a quantitative and qualitative viewpoint (Fanzo et al., 2018). For these reasons, we are forced to develop new cropping systems to be able to respond to the growing global demands for plant foods and related nutrients, but in terms of greater sustainability and resilience toward the environmental stressors (Rivero et al., 2021). In this context, grafting is one of possible solutions to alleviate the negative response of horticultural crops to environmental cues, generating a multitude of genetic/metabolic effects, substantially modifying the bioagronomic response of many fundamental crops for human nutrition (Allevato et al., 2019). Grafting is a vegetative propagation technique which connects together two different plant partners (the scion and the rootstock), thus generating a chimera carrying separately selected characteristics of root and shoot genotypes. This technique is known since ancient times in woody crops. In nature, spontaneous root grafts have been reported in about 200 species (Graham and Bormann, 1966), but they probably exist in thousands of other ones (reviewed by Lev-Yadun, 2011). By the end of the twentieth century, grafting had gained popularity in many horticultural systems worldwide, including herbaceous crops (solanaceous and cucurbits) grown under a wide array of cultivation conditions, both in open field or greenhouse (Fullana-Pericàs et al., 2020). Nowadays, the graft-induced modifications of scion phenotype are well documented for a multitude of horticultural crops, including pivotal traits such as yield potential (Mauro et al., 2020b), canopy traits (Zhu et al., 2020), fruit ripening period (Burak et al., 2008), tolerance to non-optimal thermal regimes (Singh et al., 2020; Gisbert-Mullor et al., 2021), drought or root hypoxia (Mauro et al., 2020a; Prinsi et al., 2021), mineral toxicity (Cangi et al., 2020), soil nutrient deficiency or salinity (Nawaz et al., 2016; Semiz and Suarez, 2019), along with tolerance/resistance to plant pathogens and pests (Haroldsen et al., 2012). Thus, in the view of increasing yield stability and crops' resilience in future agriculture, it is to be expected that grafting will have increasing importance, as it helps also to combine different desirable traits addressing multiple stressors, without the undesirable combinatorial or pleiotropic effects often generated by breeding programs (Rouphael et al., 2018). Furthermore, grafting can be used as a means to modulate the compositional traits of the horticultural products, including primary and secondary metabolite concentrations (Kyriacou et al., 2017; Mauro et al., 2020c). Such functional interdependence involves a complex, bidirectional communication network among the two plant partners, including the exchange of water, nutrients, hormones, peptides, nucleic acids, or other metabolites (Albacete et al., 2015), according to mechanisms whose physiology and determinism remain largely unknown. These interests have stimulated us to edit this Research Topic, collecting a total of 23 contributions (6 reviews and 17 original research articles) which cover different *Physiological and Molecular Aspects of Plant Rootstock-Scion Interactions*. In particular the topics cover rootstocks effects on scion development, including both abiotic and biotic stress tolerance/resistance, as well as the potential molecular mechanisms involved, the use of transgenic rootstocks and the causes of poor grafting success or graft incompatibility; one paper also discuss the innovative techniques that have been used in the model species *Arabidopsis thaliana*. A discussion of these articles is given below.

## Grafting Techniques

The use of plant grafting in science is accelerating in part due to the innovative techniques developed for the model plant *A. thaliana* (Bartusch and Melnyk). The authors reviewed these developments and discussed advantages and limitations associated with grafting various *A. thaliana* tissues at diverse developmental stages.

## Graft Union Formation and Grafting Success

Grafting success and graft compatibility are essential to use rootstocks on a commercial scale. Therefore, it is important to predict possible incompatibility problems at an early growth stage in order to ensure long-term survival of scion/rootstock combinations. Protein, hormones, mRNA and small RNA transport across the graft junction is currently emerging as an important mechanism controlling the rootstock-scion communication and simultaneously may play a crucial role in understanding the physiology of grafting. In their review, Rasool et al. provide an understanding of the physiological, biochemical and molecular basis underlying grafting, with special reference to horticultural plants. The understanding of molecular mechanisms underlying the affinity between graft partners can help to overcome compatibility problems, expanding the opportunities offered by the genetic biodiversity. By using a peach/plum grafting model, a couple of PAL genes codifying for phenylalanine ammonia-lyase, involved in phenylpropanoid pathway, have been identified by Amri et al. as potential markers of the translocated graft-incompatibility among those species, which often appears in the scion several years after grafting.

Loupit and Cookson review current knowledge of the changes in metabolism and transcript abundance during graft union formation and in cases of graft incompatibility with the view to identifying molecular markers. Incompatibility appears to be associated with secondary metabolism and growth regulating hormone metabolism, with highly species-dependent results. Prunasin in pear/quince grafts was the first identified markers of graft compatibility but other research has continued to identify other markers in *Prunus* such as UDP-glucose pyrophosphorylase (UGPase) activity. In other species such as grapevine, stilbenes and some flavanols are among the potential markers of graft incompatibility.

## Long-Distance Communication Between the Scion and the Rootstock

Up to now, the graft-induced systemic changes and long-distance signaling between graft partners have mainly been investigated in model plants, such as *Arabidopsis thaliana* and *Nicotiana benthamiana*. However, these aspects vary among species, so the study on model plants provides only theoretical reference for researches on grafted vegetables. In their review, Lu et al. summarize recent progress on some physiological aspects related to grafted vegetables, particularly focusing on long-distance molecular signaling (e.g., RNA and proteins). The authors provide a theoretical basis for studies of the rootstock–scion interaction in vegetables, as well as guidance for rootstock breeding to meet specific demands for efficient production.

Kapazoglou et al. review the current knowledge on the epigenetic changes and transcriptional reprogramming associated to rootstock–scion interactions in woody species. Coding and non-coding RNAs as mobile small RNAs (siRNAs) have been identified migrating across the graft union, mediating gene expression. Modifications in the DNA methylation patterns of the recipient partner have been reported, leading to altered chromatin structure, transcriptional reprogramming and graft performances. If these changes are heritable they can lead to stably altered phenotypes, making grafting an alternative to breeding for the production of superior plants with improved traits.

The review of Tsaballa et al. deals with the genomic interactions between grafting partners in scion's performance with a particular focus of herbaceous species. Special emphasis is given to the relation between epigenetics and the changes in morphology and quality of the products. Recent advances in plant science provide new information regarding the molecular interactions between rootstock and scion. Genetic exchange happens across grafting junctions between rootstock and scion, potentially affecting grafting-mediated effects already recorded in grafted plants. Furthermore, significant changes in DNA methylation are recorded in grafted scions, suggesting that these epigenetic mechanisms could be implicated in grafting effects.

According to Bantis et al., understanding the effect of heterografts on the chimeric whole plant responses is important, but the effects of the grafting itself should not be neglected. When comparing transcriptomics in homo and heterografts plantlets of watermelon (scion) and squash (rootstock), the highest numbers of differentially expressed genes were found between self-grafted and non-grafted plants in both species. Among these genes, only one (LOG5), involved in cytokinin biosynthesis, was common in all combinations where the squash acted as root, which could provide new cues to understand the mechanisms of these species in providing vigor to watermelon.

## Effects of Grafting on Product Quality

In the study conducted by Špika et al. the scion × rootstock interactions modified the sensory profile and volatile aroma compounds of tomatoes cv. Clarabella and Estatio when grafted onto different rootstocks. The fruits from “Clarabella” grafted onto “Buffon” and “Arnold” were found sweeter than those obtained from self-grafted “Clarabella.” Grafting “Clarabella” onto the tested rootstocks led to several changes in the volatile composition, whereas differences between the combinations with scion “Estatio” were not generally detected. This study showed the possibility of altering the composition of volatile aroma compounds and sensory properties of tomato fruits through the use of grafting.

The study conducted by Trandel et al. showed how grafting the triploid watermelon “Liberty” onto the interspecific rootstock “Carnivor” decreased the incidence of the internal disorder known as “hollow heart” (HH, i.e., the cracking of placental tissues) and contextually increased fruit tissue firmness. These positive interactions were found to be related to cell wall polysaccharide composition of the scion grafted onto the interspecific rootstock. This finding can be exploited as a useful mean for growers to manage HH in susceptible cultivars.

## Modulation of Crops' Response to Biotic Stressors

The performance of grafted snake melon in organic farming was studied by Flores-León et al. under high biotic and salt stress conditions. Soilborne diseases, combined with the incidence of some viruses (WMV, ZYMV, and ToLCNDV) and *Podosphaera xanthii* attacks, reduced yield by more than 50%. Salt stress had a minor impact, although synergic with the biotic stress. Under biotic stress, grafting consistently reduced plant mortality, but negatively affected consumer acceptability. *Cucumis* F1Pat81 rootstock minimized this side effect. Thus, the use of *Cucumis* rootstocks seems to be a strategy to enable resilient organic farming production of snake melon targeted to high-quality markets in organic farming.

In the research of Expósito et al., tomato and melon grafting onto the resistant rootstocks “Aligator” and *Cucumis metuliferus* (acc. BGV11135), respectively, did not always provide higher tolerance levels to *Meloidogyne incognita* if compared to ungrafted plants, whereas the relative crop yields detected both in spring and summer were enhanced. In detail, only nematode tolerance detected on ungrafted melon significantly differed from the grafted melon cultivated in summer (but not in spring). As regard this latter crop, the fruit quality depends on crop season.

Grafting can improve the resistance of watermelon to soil-borne diseases, but the molecular mechanism of defense is not completely understood. Zhang et al. used a proteomic approach to investigate the molecular basis involved in grafted watermelon leaf defense against *Fusarium oxysporum* f. sp. *niveum* (FON). The bottle gourd rootstock-grafted (RG) watermelons were highly resistant to FON compared with self-grafted (SG) plants. Proteins involved in signal transduction positively regulated the defense process. The disease resistance of RG watermelon seedlings may also be related to the improved scavenging capacity of reactive oxygen species (ROS). The expression profile of 10 randomly selected proteins was measured using quantitative real-time PCR, among which 7 was consistent with the results of the proteomic analysis.

Cháves-Gómez et al. screened potential rootstocks for resistance to vascular wilt caused by *Fusarium oxysporum* f. sp. *physali* (FOph) within several genotypes of *Physalis* spp., by using different physiological variables. Among the tested genotypes, *P. floridana* and *P. ixocarpa* plants inoculated with FOph showed similar behavior to non-inoculated plants, whereas *P. peruviana* “Colombia” and “Sudafrica” showed higher susceptibility to the vascular wilt, with net photosynthesis rate, stomatal conductance, leaf water potential, and Fv/Fm being lower than in the non-inoculated plants. The former genotypes can be considered in breeding programs or as rootstocks to establish cape gooseberry in FOph-infested soils.

The potential of grafting to increase resistance to Plum Pox Virus in stone fruits trees was studied by Sidorova et al. The scion “Startovaya” was grafted onto the interspecific rootstock “Elita.” Transgenic lines of both cultivars showed elevated siRNA levels, blocking the movement of the virus through the GM tissues into the non-transgenic partner, after inoculation of transgenic tissues. The mobile siRNA was not moved from the GM rootstock to the target non-transgenic tissue to a sufficient level to trigger silencing of PPV. This study showed that transgrafting in fruit trees remains an unpredictable practice, needing further research.

## Modulation of Crops' Response to Abiotic Stressors

Other articles have addressed how different rootstocks can improve also tolerance to non-optimal growth conditions. Bristow et al. exposed the tomato cultivar BHN-589, either ungrafted or grafted onto four commercial rootstocks, to optimal (24°C) and suboptimal soil temperature (SST, 13.5°C). Under SST, root hydraulic conductivity and conductance, stomatal conductance (gs) and plant biomass decreased, but only two rootstocks maintained higher gs. All phenotypes showed greater reductions in shoot biomass than root biomass resulting in greater (~20%) root-to-shoot ratios. Some commercial rootstocks maintained better rates of stomatal conductance and shoot N content, thus contributing to better performance under SST.

In the research paper of Fu et al., cold-tolerant pumpkin (Cm) rootstock enhanced the chilling tolerance of grafted cucumber. Chilling stress increased the endogenous salicylic acid (SA) content, especially in cucumber (Cs) scions grafted onto Cm rootstocks. These changes were linked to the up-regulation of key genes of SA biosynthesis. The accumulation of SA in the Cs/Cm leaves was mainly attributed to an increase in SA biosynthesis in leaves and that in transport from roots under aerial and root-zone chilling stress, respectively. In addition, exogenous SA significantly upregulated the expression level of cold-responsive (COR) genes, and physiological activities linked to cold tolerance.

He et al. analyzed the responses of different scion/rootstocks combination of apple to cadmium and found that the metal accumulation and tolerance differed among graft combinations. The authors show that the rootstock can modify the cadmium tolerance of the trees, potentially by restricting its accumulation and translocation. They suggest that the root cell walls of the cadmium tolerant rootstocks immobilized cadmium, reduced foliar oxidative stress markers and increase foliar antioxidant capacity, but that these responses interact with the scion genotype.

## Breeding for Rootstock and Scion Traits

Through the use of SSR genetic markers Reyes-Herrera et al. demonstrates the rootstock mediated narrow-sense inheritance of some complex plant traits in the scion of avocado trees. Beyond the root phenotype, significant heritability values were found for traits related to vigor and fruit harvest and quality, which is very relevant for rootstock breeding programs in woody and vegetable species.

Pina et al. present the first report of quantitative trait loci associated with graft incompatibility in any plant species using an F1 apricot scion mapping population grafted onto a unique *Prunus* rootstock. They characterize anatomical features of the graft interface associated with grafting success, such as the necrotic line, bark and wood discontinuities, as well as an overall assessment of the completeness of the graft union development. Two genomic regions were associated with graft incompatibility. This knowledge could aid in the identification of genes regulating graft union formation and provide knowledge to apricot breeding programs.

## Influence of Grafting on Crops' Agronomic Performances

Kviklys and Samuoliene reported a description of the relationships between rootstock, crop load, soluble sugars and hormones in two apple tree cultivars and their effects of biennial fruit bearing. The authors found that return to bloom was dependent on rootstock, scion and crop load, and was negatively correlated to sorbitol in the buds. The ratio among promoting to inhibiting hormones concentrations was only correlated with return to bloom in one scion cultivar, suggesting that apple cultivars differently respond to hormonal cues related to return to bloom.

Rossdeutsch et al. studied nitrogen uptake and transport in two graft combinations of grapevine, conferring different vigor to the scion. The low vigor rootstock had higher nitrate uptake capacity and assimilation in roots after nitrate resupply than the high vigor rootstock, which is potentially linked to the higher carbohydrate status of the low vigor rootstock. However, the high vigor rootstock had the greatest capacity to transfer nitrogen to the scion, a feature that which was potentially due to its higher transpiration rate.

Understanding the effects on fruit traits is essential for developing new commercial rootstocks. Pumpkin is the most common rootstock used in watermelon to overcome soilborne diseases, but some graft combinations can affect fruit development and quality, such as delays in ripening. In the paper of Guo et al. fruit transcriptomics analysis revealed important differences in gene expression between pumpkin-grafted and self-grafted watermelon plants, highlighting a particular impact in ABA-signaling, sucrose transport and carotenoid metabolism related genes associated to the ripening process affected by the pumpkin rootstock.

## Conclusions

Nowadays, grafting is successfully practiced in many countries around the world, closely linked to the success of many crops. Since the nineteenth century, the mostly empirical evolution of this technique has allowed the survival of entire woody crops, as in the case of citrus orchards or vineyards. Since the end of the twentieth century, grafting has also evolved in herbaceous cultivation systems, in parallel with the scientific interest in understanding the physiological and molecular processes involved, thus contributing to the growing demands for securing a sustainable food production. From a practical viewpoint, this success was largely based on the enormous benefits brought by grafting (e.g., resistance to pathogens and pests, increased yields, modulation of the production periods) overcoming the drawbacks linked to increased costs (e.g., related to seeds, skilled labor, facilities and equipment to rise grafted plants).

Modern challenges prompt agriculture toward the development of environmentally-sound systems, able to produce more nutrient-dense foods, in a context of increasing depletion of natural of resources and ecosystems vulnerability. In this context, grafting has the chance to give a pivotal contribution, provided that it will allow to benefit crops in environments with increasingly marginal characteristics. In this view, the most recent advances in physiological, biochemical, molecular and -omic studies are expected to significantly contribute toward interpreting and managing the multifaceted nature of plant scion-rootstock interactions. This is a complex and compelling challenge, whose outcome will influence the ability of agriculture to improve the genetic and agronomic potential of many fundamental crops for human nutrition, within a framework of greater sustainability.

## Author Contributions

All authors listed have made a substantial, direct, and intellectual contribution to the work and approved it for publication.

## Conflict of Interest

The authors declare that the research was conducted in the absence of any commercial or financial relationships that could be construed as a potential conflict of interest.

## Publisher's Note

All claims expressed in this article are solely those of the authors and do not necessarily represent those of their affiliated organizations, or those of the publisher, the editors and the reviewers. Any product that may be evaluated in this article, or claim that may be made by its manufacturer, is not guaranteed or endorsed by the publisher.
